# Efficacy and safety of sedation induced with cipepofol alone versus a dexmedetomidine plus propofol regimen for patients requiring mechanical ventilation: a randomized, open-label, non-inferiority trial

**DOI:** 10.1016/j.aicoj.2026.100147

**Published:** 2026-09-01

**Authors:** Caihong Yan, Hui Ouyang, Rongfan Zhai, Bo Li, Zhijia Huang, Qunfeng Zhang, Zhengmao Wu, Xianxun Jiang, Yi Lv, Ying Zhang, Junbiao Luo, Peng Li, Zhoucheng Lu, Song Liu, Shuwu Zhong, Peng Xie, Junfa Zeng, Hong Xiao

**Affiliations:** a Department of Critical Care Medicine, The Second Affiliated Hospital of University of South China, Hengyang 421001, China; b Department of Critical Care Medicine, Zhuzhou Hospital Affiliated to Xiangya School of Medicine, Central South University, Zhuzhou 412007, China

**Keywords:** Cipepofol, Sedation, Intensive care unit, Mechanical ventilation

## Abstract

**Objective:**

This randomized, open-label, non-inferiority trial aimed to compare the safety and efficacy of cipepofol vs. a dexmedetomidine plus propofol regimen for sedating intensive care unit (ICU) patients requiring mechanical ventilation (MV).

**Methods:**

This single-center study randomly assigned Chinese adult patients to cipepofol or dexmedetomidine/propofol groups (1:1 ratio). Cipepofol was infused intravenously at a loading dose of 0.1 mg/kg (over 4 min ± 30 s) and an initial maintenance dose of 0.3 mg/kg/h, which was adjusted within the range 0.06–0.8 mg/kg/h to produce sedation in the range of 0 to −4 on the Richmond Agitation-Sedation Scale. Propofol and dexmedetomidine were infused intravenously at initial dosages of 0.3 mg/kg/h and 0.2 μg/kg/h, respectively (neither drug required a loading dose). When the maximum dexmedetomidine dose (0.7 μg/kg/h) was insufficient to achieve the target sedation depth or intolerance developed, the propofol dose was modified within the range 0.3–4.0 mg/kg/h. The primary endpoint was sedation success rate, and the non-inferiority margin was set as 10%.

**Results:**

Of 72 patients enrolled for the intention-to-treat (ITT) analysis, 71 patients were included in the full analysis set/per-protocol set (FAS/PPS). The sedation success rates for the cipepofol and dexmedetomidine/propofol groups were 100% and 97.2%, respectively, in the ITT analysis and 100% for both in the FAS/PPS. Non-inferiority was established, and the lower limit of the 95% confidence interval (CI) for the inter-group difference was −1.34% for the ITT analysis and −9.64% for the FAS/PPS. There were no differences between groups in sedation compliance rate, extubation and recovery times, MV duration, or length of ICU stay (all *P* > 0.05). Cipepofol was associated with lower incidences of treatment-emergent adverse events (TEAEs; 33.3% vs. 66.7%) and drug-related TEAEs (30.6% vs. 63.9%) than dexmedetomidine/propofol (all *P* < 0.05). Notably, hypotension incidence was lower in the cipepofol group than in the dexmedetomidine/propofol group (30.6% vs. 61.1%, *P* = 0.017).

**Conclusion:**

Cipepofol was not inferior to dexmedetomidine/propofol for >24-h sedation in Chinese adult patients receiving MV in the ICU. Furthermore, cipepofol elicited fewer hypotensive events than dexmedetomidine/propofol. Large-scale, multicenter studies are warranted to confirm our findings.

## Introduction

Mechanical ventilation (MV) is frequently employed in the intensive care unit (ICU), but seriously ill patients usually require sedation and analgesia to be able to tolerate this supportive therapy. Updated guidelines released in 2018 recommend either dexmedetomidine or propofol rather than midazolam as the preferred sedative for ICU patients, since midazolam is associated with increased risks of postoperative delirium and ICU-acquired weakness as well as a longer MV duration [1]. Propofol is a widely utilized short-acting intravenous anesthetic characterized by a rapid onset and short half-life [2]. Nevertheless, higher doses of propofol can exert substantial inhibitory effects on the circulatory and respiratory systems via actions on the central nervous system, leading to unwanted symptoms such as hypotension and respiratory depression [3]. In addition, hypertriglyceridemia and propofol infusion syndrome have been reported following long-term infusion of propofol [4,5]. Dexmedetomidine, a selective α_2_‑adrenoreceptor agonist, has gained prominence in recent years as an adjunctive, but not primary, sedative agent in the ICU setting [6,7]. One reason for this is that dexmedetomidine elicits agitation in some patients when used alone [8]; indeed, one study reported that approximately 64% of ICU patients administered dexmedetomidine also required the adjunctive use of propofol to achieve adequate sedation [9]. A notable advantage of concomitant therapy is that lower dosages of each drug (opioid and sedative) are needed, which results in a reduction in adverse effects (e.g., delirium, postoperative nausea and vomiting, and ventilator‑associated pneumonia) compared to either drug administered alone [10,11]. However, one study found that the adjunctive use of dexmedetomidine with propofol did not significantly reduce MV duration, length of ICU stay, or incidence of delirium versus propofol alone [12]. Additionally, a retrospective analysis observed a higher risk of hypotensive events for concomitant dexmedetomidine and propofol than for either agent alone [13]. Consequently, the selection of appropriate sedative drugs and combination regimens is of paramount importance for the prognoses of critically ill patients.

Cipepofol (former name: ciprofol), a propofol analog with a single diastereoisomer and two chiral centers in the R-configuration, has a four-to-five-fold higher potency than propofol [14]. Previous Phase II/III trials found that cipepofol was not inferior to propofol in terms of sedation profile [15,16], and cipepofol was approved for the sedation of patients undergoing MV in the ICU in July 2022. Post-hoc analyses of ICU patients requiring sedation for 6–24 h revealed that the incidence of hypotension was lower for cipepofol than for propofol when the target light sedation goal was achieved; consequently, the short-term prognosis was more favorable for cipepofol than for propofol [17]. Moreover, when compared with propofol, cipepofol exhibited a comparable moderate time (>24 h) sedation success rate, elicited fewer hypotensive events, and was associated with a lower demand for vasopressors in critically ill patients undergoing MV, when the target sedation goal was +1 to −4 on the Richmond Agitation-Sedation Scale (RASS) [18]. However, there is a lack of studies comparing cipepofol with dexmedetomidine/propofol combination regimens for moderate time (>24 h) sedation of patients in the ICU setting.

Therefore, we conducted a randomized, open-label, non-inferiority trial to compare the safety and efficacy of cipepofol alone versus combination therapy with dexmedetomidine and propofol for the sedation of adult patients undergoing MV in China. The primary hypothesis was that cipepofol was not inferior to dexmedetomidine plus propofol with regard to the sedation success rate and other sedative effects in Chinese ICU patients receiving MV. We also tested the secondary hypothesis that cipepofol alone produced fewer hypotensive events than concomitant dexmedetomidine and propofol.

## Methods

### Study design and patient enrollment

This study was conducted at the Second Affiliated Hospital of University of South China from January 2023 to April 2023. Adult patients undergoing MV in the ICU, who required a target sedation level on the RASS of 0 to −4, were assigned randomly in a 1:1 ratio to a cipepofol monotherapy group or combination dexmedetomidine/propofol group. The present trial complied with the principles of the Declaration of Helsinki and was approved by the ethics committee of the Second Affiliated Hospital of University of South China (approval number: 20221122901). Written informed consent was obtained from all patients or their legal representatives prior to enrollment. The trial was retrospectively registered with the Chinese Clinical Trial Registry (identifier: ChiCTR2500111478) on October 31, 2025.

The inclusion criteria were: (1) adult patient aged <80 years-old; (2) body mass index (BMI) ≥18 kg/m² and ≤30 kg/m^2^; (3) tracheal intubation was required for MV; (4) expected sedation duration of at least 24 h following randomization; and (5) sedation target within the RASS range 0 to −4. Patients were excluded if: (1) they were known to be allergic to eggs, soy products, propofol, cipepofol, dexmedetomidine, opioids or their rescue drugs, or they had contraindications to cipepofol, propofol, dexmedetomidine, opioids or their rescue drugs; (2) they had been sedated for >3 days in the ICU before the provision of written informed consent or were postoperative patients from general wards who had been sedated for >3 days in the Anesthesia ICU (AICU) before being transferred to the ICU; (3) they had severe cardiovascular, neurologic/psychiatric, hepatic, or renal system diseases during the screening period, or the investigator considered the risks of sedation/anesthesia to be extremely high; (4) they were in a terminal state or had an expected survival period of no more than 72 h; or (5) they were pregnant or lactating women.

### Randomization and blinding

A simple randomization method was employed without stratification or blocking. A statistician not involved in any of the clinical assessments generated a random number table using SAS software, and eligible patients were randomly allocated to the cipepofol or dexmedetomidine/propofol group in a 1:1 ratio. The random allocation scheme was enclosed in sealed and opaque envelopes. Investigators opened the envelopes sequentially in the order of patient enrollment to determine the group assignment and corresponding intervention. A double-blind, double-dummy trial was not feasible owing to the particularity of dose adjustment when propofol was used in combination with dexmedetomidine. Therefore, this study adopted an open-label design, with patients and investigators all being aware of the group allocations.

### Medication and sedation procedures

The trial period comprised screening (Day −1 to Day 1 prior to drug administration), drug administration (Day 1) and follow-up (0–24 h after drug administration had ceased). When necessary, the patient was permitted to receive analgesia during the screening period before study drug administration (constant intravenous infusion of remifentanil at a loading dose of 0.5–1.0 μg/kg and a maintenance dose in the range 0.02–0.15 μg/kg/min). If sedation was required prior to the initial administration of the study drug(s), propofol was administered intravenously at a dose of 0.25–0.5 mg/kg each time, and the study drug was not initiated within 30 min after the last dose of propofol. Moreover, it was confirmed that the baseline sedation level of the patient had reached a RASS of ≥ −4 before study drug administration was initiated, to allow maintenance of the desired sedation level while avoiding excessive sedation. This study adopted a goal-directed sedation strategy that aimed to achieve sedation within the RASS range of 0 to −4, using dynamic clinical decision making to obtain a suitable sedation level for each patient. It should be noted that the RASS level of 0 was intended to represent an “ideal” clinical target state that would be conducive to minimizing the duration of MV and ICU stay, and that a RASS level of 0 was used as part of a composite criterion that also included comfort and ventilator synchrony. Thus, when a RASS level of 0 was accompanied by intolerance, pain or medical necessity (such as intracranial hypertension), the drug dosage was adjusted to deepen sedation to a lower RASS level, with analgesics added first and then sedatives.

During the drug administration period, each patient was given the appropriate study drug(s) and remifentanil for sedation and analgesia:

**Analgesia scheme:** The dose of remifentanil was maintained within the range 0.02–0.15 μg/kg/min, and the Critical Care Pain Observation Tool (CPOT) was used for the assessment of pain. The dose of remifentanil was adjusted during the maintenance period if the CPOT score was ≥3 points. Additionally, the remifentanil dose could be adjusted to achieve an appropriate level of analgesia even when the CPOT was <3 points, if the patient complained of pain, or the investigator judged the patient to be in unacceptable pain based on their vital signs.

**Sedation scheme:** Cipepofol was administered intravenously at a loading dose of 0.1 mg/kg over 4 min (± 30 s), according to the condition of the patient, and an initial maintenance dose of 0.3 mg/kg/h. The cipepofol dosage during the maintenance period was adjusted within the range 0.06–0.8 mg/kg/h to attain a sedation depth of RASS 0 to −4. Top-up doses of 0.05 mg/kg cipepofol (administered over a period of 30 s to 1 min) were permitted during the maintenance period, according to the patient’s condition, with an interval of at least 2 min between administrations. Neither dexmedetomidine nor propofol required a loading dose. Propofol and dexmedetomidine were infused intravenously at initial dosages of 0.3 mg/kg/h and 0.2 μg/kg/h, respectively, and the dose was calibrated to achieve a sedation depth of RASS 0 to −4. When the maximal dexmedetomidine dose of 0.7 μg/kg/h was insufficient or intolerance developed, the propofol dose was adjusted within the range 0.3–4.0 mg/kg/h to provide adequate sedation.

Dose adjustments were not solely dependent on the RASS target range of 0 to −4 but also followed more individualized sedation targets that took into account the patient’s condition and ventilator synchrony criteria. Each dose adjustment was within the range of 0.05–0.1 mg/kg/h for cipepofol and 0.25–0.5 mg/kg/h for propofol. Given that any change in the maintenance dose requires a period of time to take effect, the patient’s condition and instrument data were monitored for 10–30 min after each dose adjustment before a decision was made whether to maintain the current dosage or proceed with further adjustments. In cases of severe patient-ventilator asynchrony during MV (such as ineffective triggering or reverse triggering as indicated by ventilator waveforms) that were accompanied by respiratory distress and an increased respiratory rate, the drug dosage was adjusted to deepen sedation to a lower RASS level. Additionally, deep sedation was indicated for patients with severe traumatic brain injury complicated by intracranial hypertension and those with severe acute respiratory distress syndrome. When deep sedation was not necessitated, the target goal was light sedation, aiming for a RASS score of −2 to 0 based on the patient’s response and ventilator synchrony criteria. Top-up doses of 0.05 mg/kg cipepofol (cipepofol group) or 0.25 mg/kg propofol (combination group) were only administered when the patient was not adequately sedated, with an interval of at least 2 min between each administration. If a RASS score of 0 to −4 could not be maintained for ≥30 min at the highest protocol-specified maintenance dosage(s) of the study drug(s), the investigator was permitted to administer other sedatives as rescue medication. Additionally, if invasive or irritating procedures (e.g., sputum suction) were performed during the study period, the dose of remifentanil or maintenance dose of the sedative drug(s) was increased as required.

Daily sedation interruption was permitted for patients with deep sedation based on the patient’s clinical condition or the investigator’s assessment, in adherence to relevant Chinese guidelines [19]. Daily short-term discontinuation of sedative agents was implemented, and sedative therapy was resumed only after the patient demonstrated the return of three out of the following four basic command-following responses and neuromuscular movements: command-following eye opening, eye tracking, command-following fist clenching, and command-following toe movement.

The criteria for weaning were as follows: (1) resolution or elimination of the underlying cause requiring MV; (2) positive end expiratory pressure ≤5–8 cmH₂O, fraction of inspired oxygen (FiO_2_) ≤0.4–0.5, and arterial oxygen partial pressure/FiO₂ ≥150–200 mmHg; (3) hemodynamic stability; and 4) presence of spontaneous breathing capacity [20]. A 3-min spontaneous breathing trial (SBT) was used to preliminarily assess the patient’s spontaneous breathing capacity. Patients who passed the brief SBT with a rapid shallow breathing index <105 bpm/L underwent a SBT for at least 30 min. If the patient met the tolerance criteria, liberation from MV was initiated, and oxygen was administered at 2–3 L/min through a tracheal intubation catheter after weaning. For patients who failed the SBT, MV was restored [21,22], and the SBT was repeated after their condition had stabilized (with an interval of at least 24 h between assessments).

### Monitoring and assessments

The RASS was used to assess sedation depth, with the target depth set as −4 to 0. The RASS level was evaluated immediately before the start of study drug administration (baseline), every 30 min during the initial 1-h dosing period, every 2 h (± 10 min) from 1-h after dosing until the end of drug administration, and every 5 ± 1 min after cessation of drug administration until the RASS level was ≥0. For patients already on propofol sedation, propofol was discontinued at least 30 min before the administration of the study drug(s), allowing for a washout period of at least 30 min to minimize any residual propofol effects. Additionally, the baseline RASS level of these patients was assessed after completion of the washout period. If the RASS level at any planned or unplanned assessment during the drug administration period fell outside the target sedation range, subsequent RASS assessments were performed at intervals of 15 ± 3 min until the RASS level returned to the target sedation range. The CPOT was used to determine the requirement for analgesia (CPOT score ≥ 3 points). CPOT assessments were performed within 30 (± 5) min before the initial study drug administration (baseline), every 30 min from the start of study drug administration until 1 h after dosing, and then every 2 h (±10 min) until the end of study drug administration. Delirium was assessed using the Confusion Assessment Method for the Intensive Care Unit (CAM-ICU) once during the screening period and within 2 h after the patient regained consciousness [23].

### Outcomes and definitions

*Primary endpoint*: the rate of sedation success, which was defined as meeting two conditions: (1) the RASS score was within the range 0 to −4 for more than 70% of the study drug administration period [15,24]; and (2) no rescue medication was used.

*Secondary endpoints*: (1) sedation compliance rate; (2) sedation compliance duration per 60-min drug administration period; (3) extubation time; (4) recovery time; (5) MV duration; and (6) length of ICU stay. The detailed definitions of the secondary endpoints are presented in Supplementary Appendix 1.

Drug exposure and dose adjustments were evaluated. Safety indicators included the incidence and severity of treatment-emergent adverse events (TEAEs), serious AEs (SAEs), and drug-related TEAEs (defined as TEAEs that were definitely, likely, or possibly related to the experimental drugs used in the trial) (see Supplementary Appendix 2 for further details). TEAEs were coded using the system organ class (SOC) and preferred terms (PTs) of the Medical Dictionary for Regulatory Activities (MedDRA version 24.0) and graded for severity in accordance with the Common Terminology Criteria for Adverse Events (CTCAE version 5.0).

In addition to the terms described in the MedDRA, sedation-related TEAEs including hypotension (systolic blood pressure [SBP] <90 mmHg or a 20% decrease from baseline that lasted for >2 min), bradycardia (heart rate < 50 beats/min for > 2 min), tachycardia (heart rate > 120 beats/min), and respiratory depression (respiratory rate <8 breaths/min for more than 1 min) were defined in accordance with previous studies [15,17,22]. Hypotension was treated at the discretion of the investigators based on the patient’s physical condition, and the interventions included fluid therapy (i.e., plasma volume expansion) and the use of vasopressor drugs (e.g., norepinephrine, metaraminol, etc.). The occurrence of respiratory depression during the trial period was evaluated by the investigators, with the focus mainly on respiratory rate after the recovery of spontaneous respiration.

### Statistical analysis

This trial adopted a non-inferiority design, and the sample size was calculated based on the success rate of sedation in the ICU as the primary endpoint. The non-inferiority margin was set at 10% [25], and the success rate acceptable for sedation in clinical practice was considered to be > 80%. Assuming a sedation success rate of 98.5% for cipepofol and 97.8% for dexmedetomidine combined with propofol, a type I error of 0.025 (one-sided), a power of 80%, and a non-inferiority margin of 10%, the required sample size was determined to be 60 cases. Estimating a dropout rate of approximately 15%, this trial included 72 ICU patients undergoing MV, who were randomly assigned to either the cipepofol group or dexmedetomidine plus propofol group, with 36 cases in each group.

The primary endpoint (sedation success rate in ICU patients) was analyzed using the intention-to-treat (ITT) principle as well as in the full analysis set (FAS) and per-protocol set (PPS). The FAS included randomized patients who were given ≥ 1 dose of the study drug and had available efficacy data, following the ITT principle. The PPS comprised all patients in the FAS who demonstrated good adherence throughout the study period, had available primary endpoint data, and were free of major protocol deviations. The Newcombe-Wilson method was used to calculate the sedation success rate in both groups, the inter-group difference in success rate, and the corresponding 95% confidence interval (CI). If the lower limit of the 95% CI of the inter-group difference was greater than −10%, it could be inferred that cipepofol alone was not inferior to the combination of dexmedetomidine and propofol. In addition, post-hoc sensitivity analyses based on the FAS were conducted for sedation success rate, sedation compliance rate, and time spent within a target sedation range of RASS −1 to −3. Secondary endpoint analyses were based on the FAS. A linear interpolation method was used to calculate the total sedation compliance duration (in minutes) as the sum of the durations between consecutive RASS assessments for which the RASS level was within the range of 0 to −4. Missing RASS values were handled using the complete case method. The sedation compliance rate was calculated as the total sedation compliance duration divided by the total duration of study drug administration. The Hodges-Lehmann method was applied to calculate the median inter-group differences and corresponding 95% CIs for sedation compliance rate and sedation compliance duration per 60-min drug administration period. The Kaplan-Meier method was used to estimate the median and interquartile range (IQR) of the extubation time, recovery time, duration of MV, and ICU length of stay, and Kaplan-Meier curves with numbers at risk were constructed. The log-rank test was utilized for comparisons between the two groups, and hazard ratios (HRs) and corresponding 95% CIs were estimated by Breslow-Cox regression. All safety analyses were based on the safety set (SS), which included all patients who had received ≥1 dose of the study drug(s) and had post-treatment safety evaluations. Continuous variables among the baseline characteristics were compared using *t* tests or rank sum tests depending on whether they were normally distributed. Categorical variables among the baseline characteristics and TEAE incidences were compared using Fisher’s exact test. In addition, the absolute risk reduction (ARR) and between-group risk ratio (RR) for all TEAEs, drug-related TEAEs, and hypotension incidence were estimated. All statistical analyses were conducted using SAS version 9.4 software. The present study did not impute missing data. All continuous variables are given as the mean ± standard deviation (SD) or median with IQR, depending on whether they were normally distributed. Categorical variables are reported as numbers with percentages. A two-sided *P*-value ≤0.05 was deemed to be a statistically significant finding.

## Results

### Patient enrollment and baseline characteristics

**Fig. 1:**
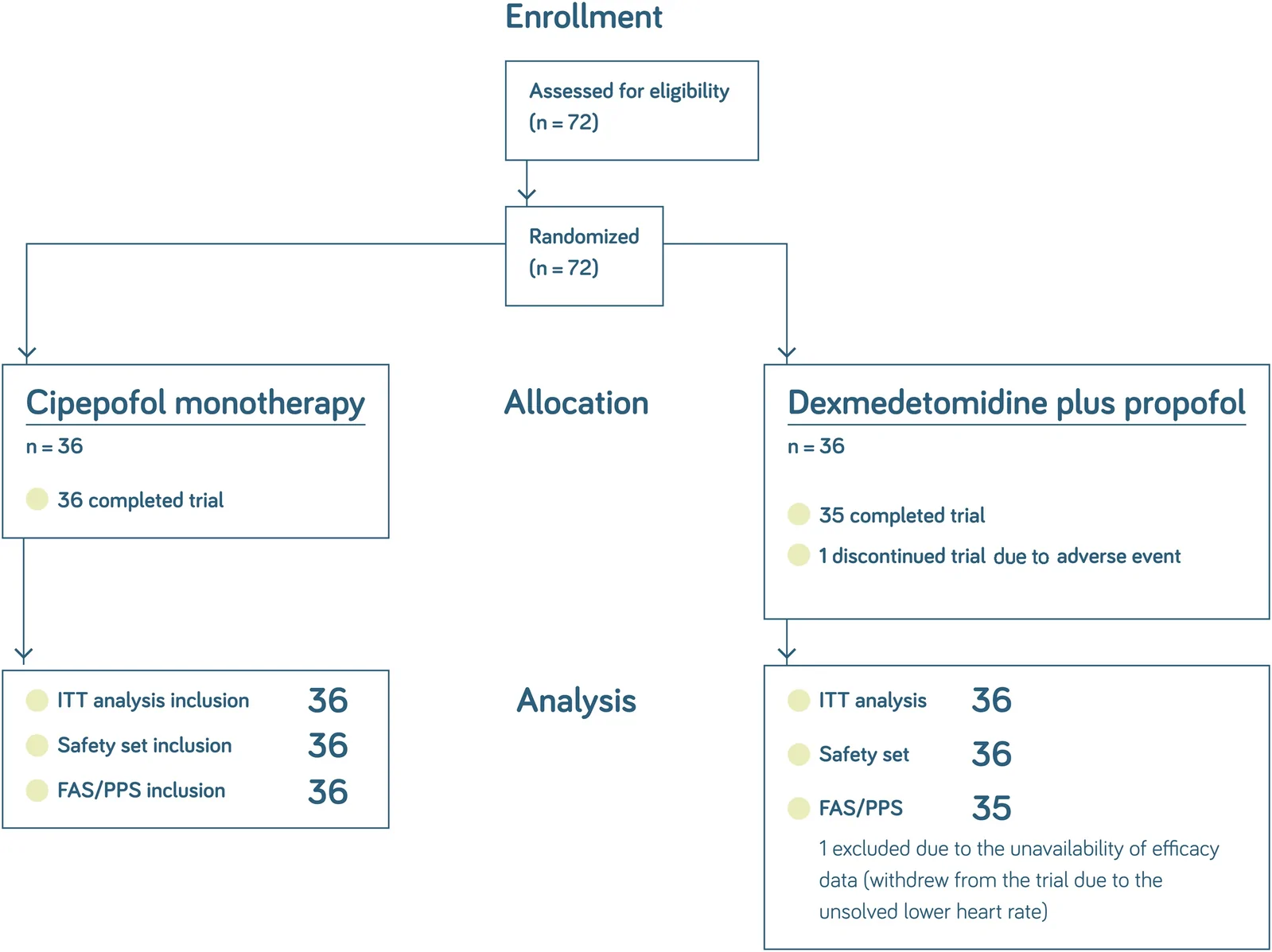
Disposition flow diagram of the patients enrolled in the trial. Abbreviations: FAS, full analysis set; ITT, intention-to-treat; PPS, per-protocol set.

All 72 ICU patients screened between January 16, 2023 and April 6, 2023 met the eligibility criteria and were randomly assigned to receive cipepofol monotherapy (n = 36) or dexmedetomidine plus propofol (n = 36) as the sedation regimen. All 72 patients were included in the ITT analysis (Fig. 1) and SS. One patient received less than 3 h of treatment with dexmedetomidine plus propofol and had no available efficacy data, and this patient withdrew from the study due to a persistently low heart rate that did not improve after discontinuation of dexmedetomidine for more than 30 min; therefore, the FAS included 71 patients. Since all patients in the FAS were free of major protocol deviations, the PPS also comprised 71 patients.

Based on the ITT analysis, median patient age was 61.5 (IQR: 55–70) years-old, with male patients (50, 69.4%) and elderly patients (46, 63.9%) predominating. The proportion of patients admitted to the ICU after surgery was numerically but not significantly higher for the cipepofol group than for the dexmedetomidine plus propofol group (80.6% vs. 63.9%, *P* = 0.114). All patients were CAM-ICU negative at baseline, and the baseline characteristics were well matched between groups (Table 1).

**Table 1:** Characteristics of the enrolled patients at baseline (ITT).

	Cipepofol (n = 36)	Dexmedetomidine plus propofol (n = 36)	*P*-value
Age (years)	63.5 (56–71)	60.0 (53.5–69)	0.493
Age range, n (%)			0.806
<65 years	12 (33.3)	14 (38.9)	
≥65 years	24 (66.7)	22 (61.1)	
Sex, n (%)			1.000
Male	25 (69.4)	25 (69.4)	
Female	11 (30.6)	11 (30.6)	
Height (cm)	170.0 (161.5–175.0)	170.0 (165.0–175.0)	0.902
Body weight (kg)	65.0 (60.7–71.3)	70.0 (60.0–75.0)	0.475
BMI (kg/m^2^)	23.1 ± 2.5	23.4 ± 2.4	0.613
Postoperative ICU admission, n (%)	29 (80.6)	23 (63.9)	0.114
Negative CAM-ICU, n (%)	36 (100)	36 (100)	1.000

Note. Continuous variables with a normal distribution were compared used the *t* test and are expressed as mean ± SD, while those with a non-normal distribution were compared with the rank-sum test and are expressed as median (interquartile range). Categorical variables were compared using Fisher’s exact test and are presented as numbers with percentages.

Abbreviations: BMI, body mass index; CAM-ICU, Confusion Assessment Method for the Intensive Care Unit; ITT, intention-to-treat.

### Primary endpoint: sedation success rate

In the ITT population, one patient withdrew from the study after receiving dexmedetomidine plus propofol for less than 3 h due to a persistently low heart rate that did not improve after discontinuation of dexmedetomidine for more than 30 min. Therefore, this patient failed to meet the criteria for sedation success. In the ITT analysis, sedation success rate was 100% (36/36) in the cipepofol group and 97.2% (35/36) in the dexmedetomidine plus propofol group, with an inter-group risk difference of 1.45% (95% CI: −1.34%, 3.24%). Sedation success rate was 100% for both groups in the FAS/PPS analysis, and the corresponding risk difference between groups was 0.00% (95% CI: −9.64%, 9.89%). Since the lower limit of the 95% CI of the median difference was greater than −10% in both the ITT and FAS/PPS analyses, it was concluded that cipepofol monotherapy was not inferior to the combination of dexmedetomidine plus propofol (Table 2). However, the post-hoc sensitivity analysis failed to support the non-inferiority conclusion under a narrower target sedation range of RASS −1 to −3: the sedation success rate did not differ significantly between the cipepofol and dexmedetomidine plus propofol groups (52.8% vs. 62.9%), but the lower limit of the 95% CI of the difference in sedation success rate was below the pre-specified non-inferiority margin of −10% (inter-group risk difference: −9.80%; 95% CI: −32.36%, 12.76%; Supplementary Table S1). Therefore, given that the primary endpoint was insufficiently discriminative in the broad target sedation range of RASS 0 to −4, the non-inferiority results should be interpreted as exploratory.

**Table 2:** Efficacy endpoints for the enrolled patients.

	Cipepofol (n = 36)	Dexmedetomidine plus propofol (n = 35)	Difference (95% CI)
Primary endpoint: sedation success rate, n (%)			
Per ITT analysis	36 (100)	35 (97.2)	1.45% (−1.34%, 3.24%)
Per FAS/PPS	36 (100)	35 (100)	0.00% (−9.64%, 9.89%)
Secondary endpoints (FAS)			
Sedation compliance rate (%)	98.0 (96.0–100.0)	98.0 (84.0–100.0)	0.00% (−1.14 × 10^−5^%, 3.56 × 10^−5^%)
Sedation compliance duration per 60-min drug administration period (min)	59.0 (58.0–60.0)	59.0 (50.0–60.0)	0.00 (−3.42 × 10^−5^, 3.04 × 10^−5^)
Proportion of time spent within the following RASS range (%)			
RASS 0 to −2	48.3 (3.6–90.5)	73.9 (45.7–99.3)	−16.22% (−42.28%, −0.38%)
RASS −3 to −4	39.3 (1.6–87.5)	14.7 (0.0–38.7)	15.14% (0.00%, 36.96%)

Note. Continuous variables with a non-normal distribution are presented as median (interquartile range), and categorical variables are presented as numbers with percentages.

Abbreviations: CI, confidence interval; FAS, full analysis set; ITT, intention-to-treat; PPS, per-protocol set; RASS, Richmond Agitation-Sedation Scale.

### Secondary endpoints

There were no significant differences between the cipepofol and dexmedetomidine plus propofol groups in sedation compliance rate (98.0% vs. 98.0%), with a median inter-group difference of 0.00% (95% CI: −1.14 × 10^−5^%, 3.56 × 10^−5^%; Table 2). Compared to the dexmedetomidine plus propofol group, the cipepofol group spent significantly less time at a sedation level of RASS 0 to −2 (median [IQR]: 12.4 [1.7–22.6] h vs. 22.5 [11.2–24.4] h, *P* = 0.009) and significantly more time at a sedation level of RASS −3 to −4 (10.1 [0.4–23.8] h vs. 5.0 [0.0–12.3] h, *P* = 0.032; Supplementary Fig. S1). Accordingly, the proportion of time spent within the RASS range of 0 to −2 was 48.3% (median value) for the cipepofol group and 73.9% for the dexmedetomidine plus propofol group (Table 2). A post-hoc sensitivity analysis revealed that the median time spent within a narrower target sedation range of RASS −1 to −3 was comparable between the cipepofol and dexmedetomidine plus propofol groups (median [IQR]: 22.6 [10.7–24.6] h vs. 21.3 [15.8–24.3] h, *P* = 0.487; Supplementary Table S1). The sedation compliance rate was 80.8% (IQR: 25.3%–98.5%) in the cipepofol group vs. 85.6% (IQR: 56.6%–98.6%) in the dexmedetomidine plus propofol group, corresponding to a median inter-group difference of −1.29% (95% CI: −22.12%, 2.88%).

**Fig. 2:**
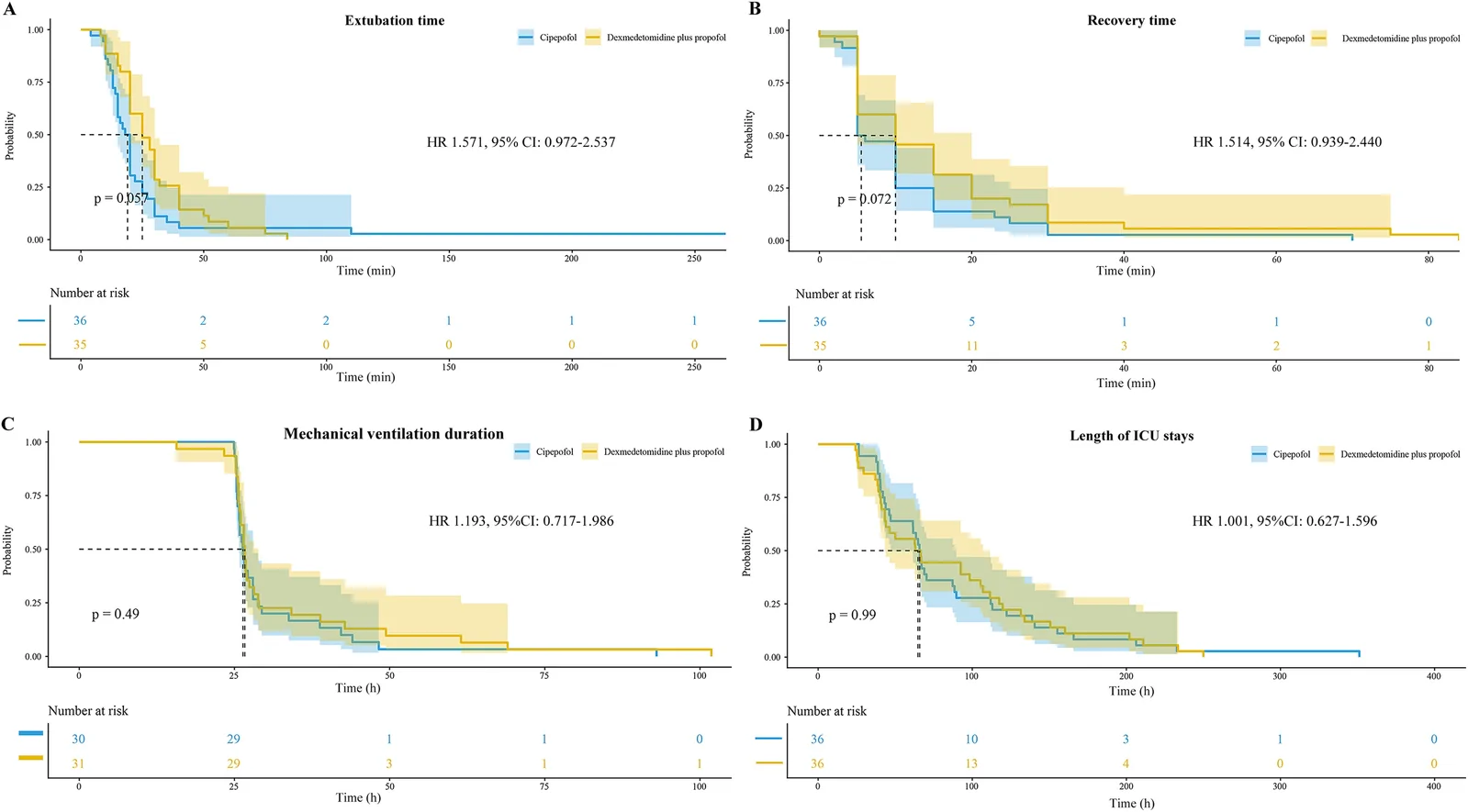
Kaplan–Meier curves of (A) extubation time, (B) recovery time, (C) mechanical ventilation duration and (D) length of ICU stay for patients receiving cipepofol alone or dexmedetomidine plus propofol. The log-rank test was used for comparisons between the two groups. Hazard ratios (HR) and corresponding 95% confidence intervals (CIs) were estimated by Breslow-Cox regression. ICU, intensive care unit.

The median extubation time was 19.0 (IQR: 13.0–25.0) min in the cipepofol group vs. 25.0 (IQR: 20.0–40.0) min in the dexmedetomidine plus propofol group, corresponding to a non-significant median reduction of 6.0 min (log-rank *P* = 0.057; Fig. 2A). The median recovery time was 5.5 (IQR: 5.0–12.5) min in the cipepofol group vs. 10.0 (IQR 5.0–20.0) min in the dexmedetomidine plus propofol group, corresponding to a non-significant median reduction of 4.5 min (log-rank *P* = 0.072; Fig. 2B). Although not reaching conventional statistical significance, the HRs of 1.571 (95% CI: 0.972–2.537) and 1.514 (95% CI: 0.939–2.440) for extubation time and recovery time, respectively, suggested a trend toward a clinically relevant benefit in the cipepofol group. There were no significant differences between the cipepofol and dexmedetomidine plus propofol groups in MV duration (median [IQR]: 26.4 [25.4–31.6] h vs. 26.8 [25.7–34.2] h, log-rank *P* = 0.490) or length of ICU stay (median [IQR]: 65.9 [42.5–112.5] h vs. 66.6 [40.6–119.7] h, log-rank *P* = 0.990) (Fig. 2C–D), and the corresponding HRs were 1.193 (95%CI: 0.717–1.986) and 1.001 (95%CI: 0.627–1.596), respectively.

In the ITT population, RASS assessment was performed 1,298 times during the drug administration period (647 times in the cipepofol group and 651 times in the dexmedetomidine plus propofol group), and the median RASS level was −2 (range: −4 to +2) in the cipepofol group and −2 (range: −4 to +3) in the dexmedetomidine plus propofol group. Baseline RASS level was ≥0 in 33 patients (91.7%) in the cipepofol group and 35 patients (97.2%) in the dexmedetomidine plus propofol group (Supplementary Fig. S2A). In the cipepofol group, one patient who had undergone embolization of an intracranial aneurysm (accompanied by intracranial hypertension) and one patient with pulmonary infection and respiratory failure required deep sedation and had baseline RASS levels of −4; the former had received propofol sedation before initiation of the study drug. The three patients in the dexmedetomidine plus propofol group who had been exposed to propofol before randomization had baseline RASS scores of −2, 0, and +2, respectively. The median RASS level at baseline was +1 for both groups, and the median RASS level after 30 min of sedation decreased to −3 in the cipepofol group and −2 in the combination group (Supplementary Fig. S2B). The slightly deeper sedation depth for the cipepofol group persisted for 11 h after sedation (median RASS: −3 vs. −2), but the sedation depth during the subsequent administration period was comparable between groups (median RASS: −2 vs. −2).

### Drug exposure

The total duration of drug administration was comparable between the cipepofol and dexmedetomidine plus propofol groups (median [IQR]: 25.0 [24.2–29.6] h vs. 24.8 [24.3–27.0] h, *P* =  0.789). The mean (SD) unit infusion rates of cipepofol, dexmedetomidine, and propofol were 0.18 (± 0.08) mg/kg/h, 0.61 (± 0.09) μg/kg/h, and 0.41 (± 0.21) mg/kg/h, respectively. The proportion of patients requiring dose adjustments during the maintenance period was significantly lower for the cipepofol group than for the combination regimen (44.4% vs. 80.0%, *P* =  0.007). The unit-time infusion rate of remifentanil did not differ significantly between the cipepofol group and dexmedetomidine plus propofol group (median [IQR]: 0.08 [0.05–0.15] μg/kg/min vs. 0.06 [0.04–0.13] μg/kg/min, *P* =  0.193; Supplementary Table S2).

### Safety outcomes

Twelve patients (33.3%) experienced 17 TEAEs in the cipepofol group, while 24 patients (66.7%) experienced 34 TEAEs in the dexmedetomidine plus propofol group. The incidence of TEAEs was significantly lower in the cipepofol group than in the dexmedetomidine plus propofol group (absolute risk difference: −33.3%, 95% CI: 10.2%–56.4%, *P* = 0.009), indicating that a number needed to treat of 3 achieved a significant benefit-risk profile (Table 3**)**. The RR was 0.50 (95% CI: 0.30–0.84), confirming a statistically robust safety advantage in the cipepofol group. Similarly, the incidence of drug-related TEAEs was significantly lower in the cipepofol group than in the dexmedetomidine plus propofol group (30.6% vs. 63.9%; RR: 0.49, 95%CI: 0.29–0.84, *P* = 0.009). No SAEs occurred, and all TEAEs and drug-related TEAEs were grade 1 or 2 in severity in both groups. Only one patient in the dexmedetomidine plus propofol group withdrew from the trial because of bradycardia that did not resolve after discontinuation of dexmedetomidine for more than 30 min.

**Table 3:** All TEAEs in the safety set.

	Cipepofol (n = 36)	Dexmedetomidine plus propofol (n = 36)	Effect size	*P*-value
Any TEAEs, n (%)	12 (33.3)	24 (66.7)	RR 0.50 (95% CI: 0.30, 0.84) ARR 33.3% (95% CI: 10.2%, 56.4%)	0.009
Hypotension	11 (30.6)	22 (61.1)	RR 0.50 (95% CI: 0.29, 0.87) ARR 30.6% (95% CI: 7.5%, 53.6%)	0.017
Tachycardia	2 (5.6)	3 (8.3)		1.000
Respiratory depression	0	2 (5.6)		0.493
Bradycardia	1 (2.8)	1 (2.8)		1.000
Elevated blood pressure	0	1 (2.8)		1.000
Any drug-related TEAEs, n (%)	11 (30.6)	23 (63.9)	RR 0.49 (95% CI: 0.29, 0.84) ARR 33.3% (95% CI:10.3%, 56.4%)	0.009
Hypotension	11 (30.6)	22 (61.1)	RR 0.50 (95% CI: 0.29, 0.87) ARR 30.6% (95% CI: 7.5%, 53.6%)	0.017
Respiratory depression	0	2 (5.6)		0.493
Bradycardia	0	1 (2.8)		1.000
SAEs, n (%)	0	0		
Severity of TEAEs, n (%)				
Grade 1	2 (5.6)	5 (13.9)		0.429
Grade 2	11 (30.6)	19 (52.8)		0.094
Grade 3	0	0		
Severity of drug-related TEAEs, n (%)				
Grade 1	0	4 (11.1)		0.115
Grade 2	11 (30.6)	19 (52.8)		0.094
Grade 3	0	0		
TEAEs that led to discontinuation of the study drugs, n (%)	0	1 (2.8)		1.000
TEAEs that led to withdrawal from the trial, n (%)	0	1 (2.8)		1.000

Note. Categorical variables were compared using Fisher’s exact test and are presented as numbers with percentages. The risk ratio between groups and its corresponding 95% CI was estimated by log-binomial regression.

ARR, absolute risk reduction; CI, confidence interval; RR, risk ratio; SAE, serious adverse event; TEAE, treatment-emergent adverse event.

Hypotension (grade 1 or 2 in severity) was the most common TEAE in both groups and was associated with both study drug regimens. The cipepofol group had a significantly lower incidence of hypotension than the dexmedetomidine plus propofol group (30.6% vs. 61.1%), with a RR of 0.50 (95% CI: 0.29–0.87, *P* = 0.017). Given the ARR of 30.6% (95% CI: 7.5%–53.6%), the calculated number needed to treat of 3 implied a highly favorable benefit-risk profile for cipepofol (Table 3). Moreover, the proportion of patients requiring vasopressor drugs was higher in the dexmedetomidine plus propofol group than in the cipepofol group (33.3% vs. 2.8% for norepinephrine, 19.4% vs. 5.6% for metaraminol). However, the median (IQR) duration of hypotension was comparable between the two groups (82.0 [60.0–142.0] min vs. 90.0 [60.0–180.0] min), as was the total dosage of vasopressor drugs used (Supplementary Table S3). Nevertheless, although the data suggest a lower observed incidence of hypotension and less frequent use of vasopressor drugs with cipepofol in this setting, larger, adequately powered trials are required to confirm this signal.

Neither group of patients experienced delirium or hypertriglyceridemia. There were no significant differences between groups in the mean triglyceride concentration at baseline, at 2 h after dosing cessation, or during follow-up (all *P* > 0.05; Supplementary Figure S3).

## Discussion

Shehabi et al. proposed an early goal-directed sedation (EGDS) strategy in 2013 [26], which enabled patients treated with non-benzodiazepines to reach light sedation goals (RASS range: −2 to +1) earlier unless they had contraindications, and this provided a favorable prognosis [27]. However, light sedation including the EGDS strategy did not reduce the incidence of ICU delirium [26] and was even associated with a higher incidence of agitation and prolongation of MV duration and ICU stay [28]. The introduction of the concept of early Comfort using Analgesia, minimal Sedatives and maximal Humane care (eCASH) also brought new thinking to the sedation of ICU patients, namely the implementation of organ function-oriented individualized sedation regimens [29]. The SPICE III study indicated that 40% of ICU patients who received a concomitant regimen of dexmedetomidine and propofol still required deep sedation (RASS ≤ −3) depending on the patient’s condition, despite a target level of light sedation [9]. A survey examining the implementation of sedation and analgesia in 116 Chinese EICU/ICU patients in critical condition found that over 70% of the sedation targets were set at RASS −3 to 0, and only 35.48% of cases had sedation targets between −1 and 0, suggesting that a relatively low proportion of these patients underwent light sedation [30]. Therefore, the current investigator-initiated study adopted a broader target sedation range of RASS 0 to −4, aiming to align as closely as possible with real-world sedation targets in Chinese ICUs [18,31]. However, our study did not stratify patients based on target sedation levels (deep vs. light) selected before enrollment. Furthermore, dose adjustment in the present trial was not solely dependent on the RASS target range of 0 to −4 but instead followed a more individualized sedation target that took into account the patient’s clinical condition. It should be noted that the RASS- and CPOT-based assessments and decisions regarding the adequacy of patient–ventilator synchrony were not governed by fully prespecified operational criteria, which may have introduced clinician-dependent variation in our open-label trial. Therefore, the observed between-group differences in comfort-related endpoints should be interpreted with caution, since part of the apparent effect may reflect clinician-influenced titration and outcome ascertainment rather than a pure pharmacologic difference between sedation strategies.

The present study adopted a non-inferiority margin of 10%, a pragmatic choice that reflected the broad sedation depth window of RASS 0 to −4 and took into account the potential safety benefits. For comparison, a previous Phase III trial utilized a narrower 8% margin for a light sedation goal of RASS −2 to +1 [15]. Based on historical data and clinical experience, we assumed that the sedation success rate would be 98.5% for cipepofol and 97.8% for dexmedetomidine combined with propofol [15,17]. Given that a success rate exceeding 80% is generally considered acceptable for procedural sedation in clinical practice, a relatively permissive non-inferiority margin of 10% would still provide a sedation success rate of well over 80%, which would be deemed clinically acceptable in ICU sedation practice.

One potential concern is the ceiling effect in the primary endpoint inherent to the use of a broad RASS range, as was illustrated by a sedation success rate of 100% in both study arms in the FAS/PPS. Successful sedation was defined as a composite endpoint (a RASS level between 0 and −4 for more than 70% of the administration period and no rescue sedation), but the relatively permissive threshold may have contributed to universal success in the FAS/PPS population. However, we would argue that the use of a composite endpoint that included rescue sedation may have mitigated against the ceiling effect. For example, in the situation where the RASS level is at −4 but sedation is still insufficient, patient-ventilator asynchrony or hemodynamic instability would likely necessitate the administration of rescue medications. Additionally, the initiation of daily sedation interruption for patients requiring deep sedation may have prevented cases of excessive sedation. Notably, analyses of RASS level over time indicated an adequate level of sedation in both groups, with the cipepofol group having a slightly deeper sedation depth and spending a relatively longer time at RASS −3 to −4 than the dexmedetomidine plus propofol group. The lower limit of the 95% CI of the inter-group difference in sedation success rate was −1.34% in the ITT population and −9.64% in the FAS/PPS (both greater than −10%), demonstrating non-inferiority (at a 10% margin) for the target sedation goal of RASS 0 to −4. However, a post-hoc sensitivity analysis did not support the non-inferiority conclusion within a narrower target sedation range of RASS −1 to −3, suggesting that the main result of our analysis was to some extent dependent on the definition of the endpoint. Given that the sensitivity analysis was not prespecified, the study sample size was not powered to detect effects for this endpoint under the narrower target sedation range of RASS −1 to −3. Additionally, the narrower target sedation range reduced the endpoint event rate and increased uncertainty, leading to a notably wider CI that implied limited robustness and imprecise estimation. Nevertheless, we acknowledge the need for further clinical studies using less broadly defined primary endpoints to confirm the non-inferiority of cipepofol vs. dexmedetomidine plus propofol.

It is worth noting that there is a distinction between statistical non-inferiority and true clinical equivalence. An inclusion criterion for this study was a required sedation depth of RASS 0 to −4, but with the exception of a few postoperative patients such as those with brain disorders who required deep sedation, the majority of patients had relatively mild conditions as indicated by the RASS distribution, MV time and duration of ICU stay. The inclusion of a relatively narrow patient population limits the generalizability of our findings, especially for hemodynamically fragile or severely ill ICU patients with serious medical conditions (cardiovascular, neurologic/psychiatric, hepatic or renal system diseases), but also for those older than 80 years-old, with a BMI outside the range 18–30 kg/m^2^, or with an expected survival period of no more than 72 h. Hence, the safety and efficacy of cipepofol in these higher-risk groups remain to be established. Another distinction was that the non-inferiority findings of this study were predicated on a sedation strategy that used dexmedetomidine as the primary agent with supplementary propofol titration, which differs from the clinically prevalent propofol-based sedation paradigm. This study did not utilize a loading dose of dexmedetomidine, in accordance with a previous study [9]. An adequate sedation level could be achieved with the lowest dose of propofol in combination with dexmedetomidine, as indicated by the RASS curve. This combination sedation strategy takes into account the pharmacologic effects of dexmedetomidine and the eCASH concept that aims to minimize the dosage of propofol and propofol-related side effects [32]. However, this limits the comparability between cipepofol and propofol. Furthermore, dexmedetomidine-based regimens require more frequent RASS assessments and more timely decisions on supplementary propofol administration in the resource-limited ICU setting. The present study also underscores the procedural complexity inherent to this combination sedation regimen, as evidenced by the significantly higher frequency of dose adjustments observed in the combination group than in the cipepofol group. These findings suggest that this dexmedetomidine-based regimen imposes greater demands on an interdisciplinary healthcare team in clinical practice, and its effectiveness is highly dependent on the implementation environment.

Previous studies have shown that the adjunctive use of dexmedetomidine was associated with earlier extubation and recovery, as the depth of sedation induced by the combination regimens was lighter than that achieved with single sedative agents [6,7]. The cipepofol group appeared to exhibit trends toward shorter extubation and recovery times, although statistically significant differences were not attained. We speculate that the non-significant *P*-values likely reflect limited statistical power attributable to the small sample size, rather than a true absence of treatment effect. However, any potential benefit in shortened extubation time was not accompanied by a reduction in overall ICU length of stay, which might reflect the availability of ICU beds, non-respiratory complications, or insufficient trial power. Taken together, the results of our exploratory analysis suggest that cipepofol sedation for a period of at least 24 h provided comparable sedation and prognostic outcomes in mechanically ventilated patients to concomitant therapy with dexmedetomidine and propofol, but these findings warrant further investigation in adequately powered trials.

Various recent studies have focused on the increased risk of adverse hemodynamic events linked with the concurrent use of dexmedetomidine plus propofol in critically ill patients who required MV [12,13]. In accordance with previous cipepofol studies [[15], [16], [17],^33^], cipepofol monotherapy was associated with a lower incidence of TEAEs and drug-related TEAEs as well as fewer hypotension events and less frequent use of vasopressor drugs, although it should be noted that larger, adequately powered trials are required to confirm this signal. Cipepofol-related studies have been predominantly conducted in Asian populations; the findings of reduced hypotension incidence require cautious interpretation when extrapolating to non-Chinese or ethnically diverse ICU cohorts. It has been reported that cipepofol had favorable hemodynamic properties at maintenance doses up to 0.8 mg/kg/h, which may be attributed to the absence of a clear exposure-response relationship between drug exposure and safety variables, such as hypotension and bradycardia [33]. These findings might explain why patients sedated with cipepofol had a slightly deeper sedation depth, but the incidence of hypotension was still lower than that in the combination group where more patients were lightly sedated. This study did not record the data for the CPOT assessments but instead employed a sedation strategy that prioritized analgesia such that each patient had a CPOT score <3 points before the initiation of cipepofol or dexmedetomidine/propofol sedation, and the dose of remifentanil was adjusted to maintain the CPOT score at <3 points during the maintenance period. As a result, changes in the CPOT score before and after sedation were minimal in the two groups. Moreover, there was no significant difference between groups in remifentanil infusion rate normalized to body weight. These approaches minimized any potential differential impact of analgesia management on sedation and hemodynamic profiles between the two groups. To date, no randomized controlled trials have shown that light sedation reduces the incidence of delirium in comparison with deep sedation. The reduced incidence of delirium in studies using a combination of dexmedetomidine and propofol may be largely attributed to the actions of dexmedetomidine as well as the reduction in propofol consumption, but this concept remains to be determined unequivocally [12]. It is noteworthy that neither group of patients in the present study experienced delirium or hypertriglyceridemia and that triglyceride levels did not differ significantly between the two groups. However, delirium assessment was only conducted on two occasions, once during screening and once within two hours after the patient regained consciousness, raising the possibility of under-detection and outcome misclassification. Therefore, this endpoint cannot be reliably interpreted in this trial.

We acknowledge that the retrospective registration of this trial is an important limitation of our study, since retrospective registration is potentially associated with a risk of biasing results. However, it should be emphasized that the protocol of this investigator-initiated study, including the pre-defined primary endpoint, non-inferiority margin, and detailed statistical analysis plan, was finalized and approved by the ethics committee of our institution on December 29, 2022. This approval was given earlier than the date of enrollment of the first patient, which was January 16, 2023. Furthermore, we adhered strictly to the statistical analysis plan that was archived on December 19, 2022, and we did not make any modifications to the endpoints or analytical methods throughout the process of data collection and analysis. Nevertheless, we acknowledge that the retrospective registration may raise concerns about the credibility of the pre-defined non-inferiority design, particularly regarding the selection of the primary endpoint and the 10% non-inferiority margin. Therefore, the findings in this study should be considered hypothesis-generating rather than confirmatory.

Since this was a single-center, open-label study with a small sample size, the results should be interpreted with caution due to the potential for bias. However, we implemented several measures to mitigate against this concern: (1) A strict randomization method was employed, in which the random number allocation was carried out using sealed envelopes, so the investigators were only aware of the intervention after enrollment, reducing the risk of bias during the allocation of patients to the groups. (2) Although this study did not record the propofol dosage used before initiation of the study drug(s), patients who had undergone continuous sedation for more than 3 days before enrollment were excluded from the analysis. Moreover, propofol was discontinued at least 30 min before administration of the study drug(s), thereby minimizing any residual propofol effects. Only four patients required propofol sedation in advance: three patients in the dexmedetomidine plus propofol group with baseline RASS scores of −2, 0, and +2, respectively; and one patient in the cipepofol group (who had undergone embolization of an intracranial aneurysm that was accompanied by intracranial hypertension) with a baseline RASS level of −4. It should be noted that successful sedation was achieved in all four of these patients, and the sedation success rates of the two groups remained comparable following the exclusion of these patients’ data. (3) Analyses of the primary endpoint conducted for the ITT and FAS/PPS populations yielded consistent non-inferiority findings, thereby confirming the robustness of the data. However, the post-hoc sensitivity analysis failed to support the non-inferiority conclusion within a narrower target sedation range of RASS −1 to −3. Nevertheless, the notably wider CI obtained in the post-hoc analysis suggested limited robustness and imprecise estimation. From a practical perspective, open-label assessment of sedation in trials may be more in line with actual clinical practice. Our study was conducted as a pragmatic comparison of sedation strategies rather than as a strictly standardized pharmacologic comparison, and this may have contributed to the greater variability in the endpoint within the narrower sedation range.

This study has some additional limitations that affect the generalizability of our findings. First, there was a predominance of postoperative and elderly patients with relatively short sedation durations, which reflects the current status of ICU patients in our hospital. As a result, the observed associations should be interpreted with caution, and their generalizability may be limited to ICU populations with similar clinical characteristics and in similar practice settings. Second, our findings may not directly extrapolate to ICUs that rigorously target light sedation. Third, all the subjects included in this study were Chinese ICU patients, hence the findings may not be generalizable to external populations (especially non-Chinese patients) given that drug pharmacokinetics and pharmacodynamics may exhibit population-specific characteristics and race-related differences that could affect drug exposure levels, sedative effects, and hemodynamic tolerance. Fourth, an important limitation of this study is the lack of established severity scores, such as the American Society of Anesthesiologists (ASA) classification, Acute Physiology and Chronic Health Evaluation II (APACHE II), and Simplified Acute Physiology Score II (SAPS II), as well as the absence of several clinically relevant baseline variables, including admission diagnosis, surgical vs. medical ICU status, indication for MV, vasopressor use, and prior sedation exposure. These factors are likely to influence both treatment selection and clinical outcomes; therefore, their absence limited our ability to fully characterize the baseline illness severity, which may have introduced residual confounding that would limit the interpretation of inter-group comparisons. Future studies should prospectively collect standardized severity measures and key ICU baseline variables to improve confounding control, interpretability, and applicability of the findings. Large-scale studies with adequate power to investigate the long-term sedative effects of cipepofol are warranted.

## Conclusion

This randomized, open-label trial performed a pragmatic comparison of two sedation strategies. The main findings demonstrated that the sedation success rate of cipepofol alone was not inferior to that of concomitant dexmedetomidine and propofol in patients undergoing MV for at least 24 h in the ICU. Additionally, cipepofol monotherapy was associated with significantly lower incidences of TEAEs, drug-related TEAEs, and hypotensive events than the dexmedetomidine plus propofol regimen. However, the non-inferiority results should be interpreted as exploratory, given that the primary endpoint was insufficiently discriminative in the broad target sedation range of RASS 0 to −4. The robustness of this finding within a more stringent and clinically relevant sedation target range warrants further validation in multicenter trials with sufficient sample sizes. Furthermore, the absence of baseline comorbidity data, severity metrics and other clinically relevant baseline variables may have resulted in residual confounding and a limited ability to fully account for differences in baseline severity and case mix. Accordingly, our findings should be interpreted within the studied cohort rather than as definitive evidence of an independent causal relationship, and their generalizability may be limited to ICU populations with similar clinical characteristics and in similar practice settings. The small sample size, single-center open-label design, retrospective registration, short duration of sedation exposure, and absence of longer-term or patient-centered outcomes also limit the generalizability of our findings. Future large-scale, prospectively registered, multicenter trials should consider the use of more granular sedation endpoints (e.g., stratification of light vs. deep sedation targets, sedation variability, rescue sedative use, or ventilator synchrony) or patient-centered outcomes such as delirium, long-term functional status, and mortality.

## CRediT authorship contribution statement

**Caihong Yan:** Conceptualization, Methodology, Validation, Formal analysis, Investigation, Writing - Original Draft, Supervision; **Hui Ouyang:** Validation, Formal analysis, Investigation, Writing - Original Draft; **Rongfan Zhai:** Validation, Formal analysis, Investigation, Writing - Original Draft, Writing - Review & Editing; **Bo Li:** Validation, Investigation, Data Curation, Writing - Review & Editing; **Zhijia Huang:** Validation, Investigation, Data Curation, Writing - Review & Editing; **Qunfeng Zhang:** Validation, Investigation, Data Curation, Writing - Review & Editing; **Zhengmao Wu:** Validation, Investigation, Data Curation, Writing - Review & Editing; **Xianxun Jiang:** Validation, Investigation, Data Curation, Writing - Review & Editing; **Yi Lv:** Validation, Investigation, Data Curation, Writing - Review & Editing; **Ying Zhang:** Validation, Investigation, Data Curation, Writing - Review & Editing; **Junbiao Luo:** Validation, Investigation, Data Curation, Writing - Review & Editing; **Peng Li:** Validation, Investigation, Data Curation, Writing - Review & Editing; **Zhoucheng Lu:** Validation, Investigation, Data Curation, Writing - Review & Editing; **Song Liu:** Validation, Investigation, Data Curation, Writing - Review & Editing; **Shuwu Zhong:** Validation, Investigation, Data Curation, Writing - Review & Editing; **Peng Xie:** Conceptualization, Methodology, Validation, Formal analysis, Investigation, Writing - Review & Editing; **Junfa Zeng:** Validation, Investigation, Data Curation, Writing - Review & Editing; **Hong Xiao:** Validation, Investigation, Data Curation, Writing - Review & Editing.

## Consent for publication

Not applicable.

## Ethics committee approval

The present trial complied with the principles of the Declaration of Helsinki and was approved by the ethics committee of the Second Affiliated Hospital of University of South China (approval number: 20221122901). Written informed consent was obtained from all participants or their legal representatives prior to enrollment.

## Funding

This research did not receive any specific grant from funding agencies in the public, commercial, or not-for-profit sectors.

## Data availability

The study data will be available from the corresponding author upon reasonable request.

## Declaration of competing interest

The authors declare that they have no known competing financial interests or personal relationships that could have appeared to influence the work reported in this paper.
