# Supplementary material for: Efficacy and safety of sedation induced with cipepofol alone versus a dexmedetomidine plus propofol regimen for patients requiring mechanical ventilation: a randomized, open-label, non-inferiority trial
Source: Ann Intensive Care. 2026 Sep 1;16:100147. doi: 10.1016/j.aicoj.2026.100147 (PMC13579693; doi:10.1016/j.aicoj.2026.100147)
Supplement: Supplementary file 1 [file mmc1.docx]

**Efficacy and safety of sedation induced with cipepofol alone versus a** **dexmedetomidine plus propofol regimen for patients requiring mechanical ventilation: a** **randomized, open-label, non-inferiority trial**

**Caihong Yan ^a, b†*^, Hui Ouyang ^a†^, Rongfan Zhai ^a†^, Bo Li ^a^, Zhijia Huang ^a^, Qunfeng Zhang ^a^, Zhengmao Wu ^a^, Xianxun Jiang ^a^, Yi Lv ^a^, Ying Zhang ^a^, Junbiao Luo ^a^, Peng Li ^a^, Zhoucheng Lu ^a^, Song Liu ^a^, Shuwu Zhong ^a^, Peng Xie ^a*^, Junfa Zeng ^a^, Hong Xiao ^a^**

^a^ Department of Critical Care Medicine, The Second Affiliated Hospital of University of South China, Hengyang 421001, China

^b^ Department of Critical Care Medicine, Zhuzhou Hospital Affiliated to Xiangya School of Medicine, Central South University, Zhuzhou 412007, China

**^†^ These authors have contributed equally to this work and share first authorship**

**^*^Correspondence to:**

**Caihong Yan, Ph.D.**

Department of Critical Care Medicine, The Second Affiliated Hospital of University of South China, No. 35 Jiefang Avenue, Huaxin Development Zone, Hengyang 421001, China

Department of Critical Care Medicine, Zhuzhou Hospital Affiliated to Xiangya School of Medicine, Central South University, No. 116 Changjiang South Road, Tianyuan District, Zhuzhou 412007, China

Email: yancaihong@usc.edu.cn

Tel: +86-18674766697

Fax: +86-7348899900

**Prof. Peng Xie, Ph.D.**

Department of Critical Care Medicine, The Second Affiliated Hospital of University of South China, No. 35 Jiefang Avenue, Huaxin Development Zone, Hengyang 421001, China

Email: 2023020221@usc.edu.cn

Tel: +86-18984959089

Fax: +86-7348899900

**Supplementary Appendix 1: Definition of secondary endpoints**

1) Sedation compliance rate was defined as the percentage of time during the total drug administration period when the Richmond Agitation-Sedation Scale (RASS) fell within the target sedation range of 0 to -4.

2) Sedation compliance duration per 60-min drug administration period was defined as the number of minutes per 60-min interval during which the RASS score was within the target range of 0 to -4, calculated from the initial administration of the study drug to the last administration. The sedation compliance duration per 60-min drug administration period was determined as follows. First, a linear interpolation method was used to calculate the total sedation compliance duration (in minutes) as the sum of the durations between consecutive RASS measurements where the RASS level fell within the range of 0 to -4. Then, this number was divided by the total duration of study drug administration (in minutes) and multiplied by 60 to obtain the sedation compliance duration per 60-min drug administration period. If the time point at which the RASS level fell to within the target range was earlier than the start of study drug administration, the calculation started from the initiation of study drug administration. Conversely, if the RASS level remained within the target range after the end of study drug administration, the calculation was capped at the time of study drug withdrawal.

3) Extubation time was defined as the time from cessation of drug administration to successful removal of the tracheal tube.

4) Recovery time was defined as the time from the patient's recovery from a sedated state to being fully alert (RASS ≥ 0) after the end of drug administration. If RASS was ≥ 0 when drug administration was discontinued, the recovery time was recorded as 0.

5) The duration of mechanical ventilation was defined as the time from intubation (for those intubated after ICU admission) or from admission to the ICU (for those intubated prior to ICU admission) until successful removal of the tracheal tube. The initiation of sedative administration occurred either subsequent to the time of intubation or admission to the ICU.

6) Length of ICU stays was defined as the time from intubation/admission to the ICU with a tube to discharge from the ICU.

**Supplementary Appendix 2. Definitions and assessments of safety-related indicators**

**1. Criteria for determining the correlation between TEAEs and the study drugs**

| **Indicators** | **Definitely relevant** | **Probably relevant** | **Possible relevant** | **Possible relevant** | **Definitely irrelevant** |
| --- | --- | --- | --- | --- | --- |
| Reasonable chronological order | Yes | Yes | Yes | Yes | No |
| Belongs to the known reaction type of the study drugs | Yes | Yes | Yes | No | No |
| Discontinuing the study drugs can improve the situation | Yes | Yes | Yes or no | Yes or no | No |
| Re-administration of the study drugs can lead to recurrence. | Yes | ? | ? | ? | No |
| The reaction can have another explanation | No | No | Yes | Yes | Yes |

**2. The assessments of safety-related indicators**

Laboratory indicators including blood and urine routine measurements, blood biochemistry, coagulation function and physical examination were conducted during the screening and follow-up periods. Additionally, the triglyceride concentration was measured once within 30 min after the completion of the study drugs administration. Blood gas analysis was evaluated before the initial administration of the study drugs during the screening period and within 2 h after the end of administration. Electrocardiographic monitoring was performed with a 12-ECG during the screening and follow-up periods and with continuous 3-ECG monitoring during the drug administration period. Vital signs, including systolic blood pressure (SBP), diastolic BP (DBP), mean arterial pressure (MAP), heart rate, respiratory rate and blood oxygen saturation (SpO_2_), were continuously monitored throughout the entire drug administration period. Vital signs were recorded within 30 min before the first dose of the study drugs as baseline, every 30 (± 5) min from the initial dosing up to 1 h after dosing, and every 2 h (±10 min) from 1 h after dosing until the end of administration. Subsequently, vital signs were recorded within 2 h after the end of drug administration and once during the follow-up period.

**Supplementary Table 1. The post-hoc sensitivity analysis regarding the efficacy outcomes within the target sedation range of RASS from -1 to -3 (FAS)**

|  | **Cipepofol**  **(n = 36)** | **Dexmedetomidine plus propofol (n = 35)** | **Difference (95% CI)** | **P value** |
| --- | --- | --- | --- | --- |
| Sedation success rate, n (%) | 19 (52.8) | 22 (62.9) | -9.80% (-32.36%, 12.76%) | 0.474 |
| Sedation compliance rate (%) | 80.8 (25.3-98.5) | 85.6 (56.6-98.6) | -1.29% (-22.12%, 2.88%) | 0.213 |
| Time spent within the target sedation RASS range (h) | 22.6 (10.7-24.6) | 21.3 (15.8-24.3) | — | 0.487 |

Note. Continuous variables with a non-normal distribution are presented as median (interquartile range), and categorical variables are presented as numbers with percentages.

Abbreviations: CI, confidence interval; FAS, full analysis set; RASS, Richmond Agitation-Sedation Scale.

**Supplementary Table 2. The utilization of study drugs and remifentanil (safety set)**

|  | **Cipepofol**  **(n = 36)** | **Dexmedetomidine plus** **propofol (n = 36)** | ***P*-value** |
| --- | --- | --- | --- |
| Total duration of study drugs use (h) | 25.0 (24.2-29.6) | 24.8 (24.3-27.0) | 0.789 |
| Cipepofol infusion rate (mg/kg/h) | 0.18 ± 0.08 |  |  |
| Dexmedetomidine infusion rate (μg/kg/h) |  | 0.61 ± 0.09 |  |
| Propofol infusion rate (mg/kg/h) |  | 0.41 ± 0.21 |  |
| Patients with at least once dose adjustment of study drugs during maintenance period, n (%) | 16 (44.4) | 28 (80.0) | 0.007 |
| Unit infusion rate of remifentanil (μg/kg/min) | 0.08 (0.05-0.15) | 0.06 (0.04-0.13) | 0.193 |

Note. Comparison with non-normal distribution were compared using rank-sum test and expressed as median (interquartile range), while continuous variables with normals distribution were expressed as mean ± standard deviation. Categorical variables were compared using Fisher’s exact test and presented as numbers with percentages.

**Supplementary Table 3. The hypotension durations and utilization of vasopressor drugs (safety set)**

|  | **Cipepofol**  **(n = 36)** | **Dexmedetomidine plus propofol (n = 36)** |
| --- | --- | --- |
| Duration of hypotension (min) | 82.0 (60.0-142.0) | 90.0 (60.0-180.0) |
| Patients with any vasopressor drugs, n (%) | 5 (13.9) | 21 (58.3) |
| Norepinephrine | 1 (2.8) | 12 (33.3) |
| Metaraminol | 2 (5.6) | 7 (19.4) |
| Dopamine | 2 (5.6) | 2 (5.6) |
| Total dosage of following vasopressor drugs (mg) |  |  |
| Norepinephrine | 17.9 ± NA | 12.1 ± 8.9 |
| Metaraminol | 129.0 ± 73.1 | 90.8 ± 55.0 |
| Dopamine | 653.6 ± 152.0 | 777.3 ± 307.3 |

Note. Continuous variables with normal distribution were expressed as mean ± standard deviation, while expressed as median (interquartile range). Categorical variables were presented as numbers with percentages. NA, not available

**Supplementary Figure 1. The median time (in hours) spent within RASS range of 0 to -2 or -3 to -4 between cipepofol and dexmedetomidine plus propofol.** Data were presented as expressed as median (interquartile range). RASS, Richmond Agitation-Sedation Scale.

**Supplementary Figure 2. (A) The numbers of patients with RASS > 0, -2, and -4 at baseline and (B) the median RASS over time during the 24-hour treatment period with cipepofol or** **dexmedetomidine plus propofol.** RASS, Richmond Agitation-Sedation Scale

**Supplementary Figure 3. The mean triglyceride concentrations of ICU patients in both groups at baseline, 2 h after the cessation of drug administration and at follow-up.** Error bars indicated the standard deviation. ICU, intensive care unit
